# Response to ‘Commentary on Thoral et al. (2024) The relationship between mitochondrial respiration, resting metabolic rate and blood cell count in great tits’

**DOI:** 10.1242/bio.061771

**Published:** 2024-11-20

**Authors:** Elisa Thoral, Carmen C. García-Díaz, Elin Persson, Imen Chamkha, Eskil Elmér, Suvi Ruuskanen, Andreas Nord

**Affiliations:** ^1^ Lund University, Department of Biology, Evolutionary Ecology & Infection Biology, Sölvegatan 37, SE-223 62, Lund, Sweden; ^2^ Lund University, Department of Clinical Sciences, Mitochondrial Medicine, Sölvegatan 17, SE-221 84, Lund, Sweden; ^3^ University of Jyväskylä, Department of Biological and Environmental Science, Survontie 9, Jyväskylä, Finland

The commentary by Malkoc et al. highlights possible arguments explaining divergent findings of how mitochondrial metabolism in blood cells is related to whole-organism resting metabolic rate (RMR) in birds across the three studies that have addressed this relationship (Casagrande et al., 2023; Malkoc et al., 2021; Thoral et al., 2024). The authors argue that the lack of correlation between ROUTINE respiration in blood and RMR in Thoral et al. (2024), but the presence of such in their previous papers (Casagrande et al., 2023; Malkoc et al., 2021), can be explained by methodology (intact versus permeabilized blood cells), acclimation temperature, or the stress status of the individuals. In our response, we argue why the concerns expressed by Malkoc et al. should be interpreted with caution, and comment on the conclusions that can be drawn from studying both individual and mitochondrial metabolisms.

**Fig. 1.**
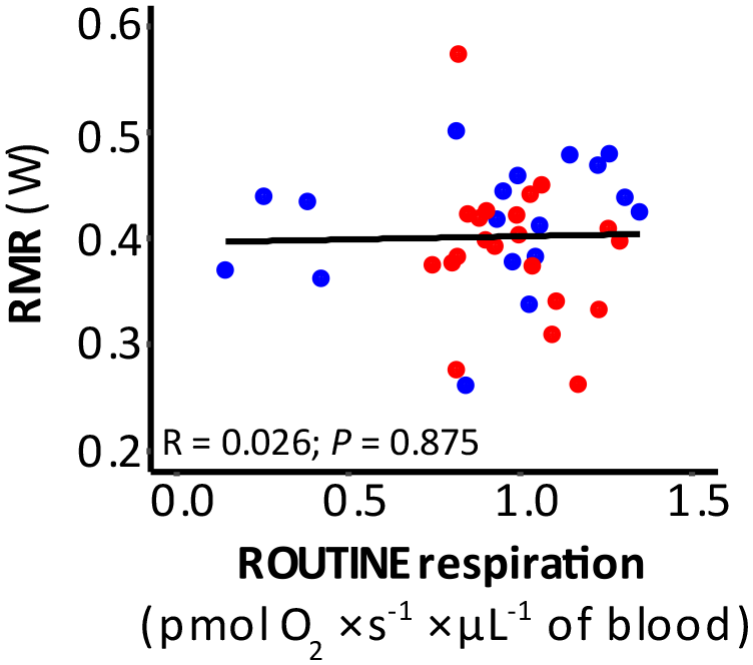
**Relationship between RMR and ROUTINE respiration obtained in intact blood cells from Japanese quail (*Coturnix japonica*).** Half of the birds were acclimated at 10°C (blue dots) while the other half was acclimated at 30°C (red dots) for 11 weeks. RMR was measured at 30°C, which is thermoneutral in Japanese quail, and blood respiration was measured at 41°C. A Pearson correlation test on pooled data revealed no significant association between RMR and ROUTINE. Nor was this relationship significant within groups (cold birds: R=0.255, *P*=0.306; warm birds: R=−0.250, *P*=0.275).

Firstly, we agree that permeabilization status can explain the absence or presence of a relationship between mitochondrial respiration traits in blood cells and RMR. However, this alone cannot explain why ROUTINE respiration was uncorrelated to RMR in Thoral et al. (2024), but correlated to RMR in Malkoc et al. (2021) and Casagrande et al. (2023), since blood cells are *not* permeabilized when ROUTINE is measured (Gnaiger, 2020). We therefore disagree that a relationship between ROUTINE respiration in intact blood cells and RMR is a general phenomenon and that the absence of such reflects neglectful experimentation. In line with this, a study on Japanese quail (*Coturnix japonica*) in our laboratory found no relationships between these traits (Fig. 1). We suggest that permeabilized experiments may bypass any rate-limiting steps influencing mitochondrial function in intact cells (Djafarzadeh and Jakob, 2017), such as substrate availability (Kyriazis et al., 2022; Leverve, 2007), transmembrane transport rate (Osellame et al., 2012), or any interactions between mitochondria and other cell components (Boldogh and Pon, 2007; Wieckowski et al., 2009). Thus, even though the measurement conditions in intact cell experiments may be more similar to the *in vivo* situation, permeabilization could be necessary to reveal a link between cellular and organismal metabolic rate. We propose that future studies apply complete SUIT protocols (substrate – uncoupler – inhibitor titration protocols; Gnaiger, 2020) in both intact and permeabilized samples from the same individual. The resultant improved understanding of mechanisms underlying phenotypic changes observed at the individual level would provide a more comprehensive ecological context.

Secondly, the authors argue that acclimation temperature may affect metabolism at different biological scales, altering the relationship between RMR and mitochondrial respiration. However, since the effect of environmental temperature on RMR is linear below the lower critical temperature (Scholander et al., 1950), it can be argued that an acute organismal metabolic response would only affect the intercept of the regression, not the slope. In support of this, the relationship between organismal metabolic rate and mitochondrial respiration parameters were largely identical when RMR was measured at 7.5°C and 20°C in our study [Fig. S4 in Thoral et al. (2024)]. Even if the timeline of changes to cellular and organismal metabolic rate differ [e.g. if acclimation time could be too short or too stochastic to cause either intrinsic changes in mitochondrial functioning or increased presence of a new blood cell phenotype (Voss et al., 2010)], it seems unlikely that variation in the temporal response would completely negate a potential underlying biological relationship. However, we agree with the incentive that “*…effort should be directed to investigate how environmental temperature, acclimatization state, body and assay temperatures affect RMR and mitochondrial metabolic rate*”. Such a study has already been performed: García-Díaz et al. (2023) exposed winter-acclimated great tits to −15°C, +5°C or +25°C for 13-15 h overnight whilst measuring RMR and body temperature, and mitochondrial respiration in intact blood cells at a normothermic (41°C) and a hypothermic (35°C) assay temperature the morning after. While RMR was nearly twice as high in −15°C compared to +25°C, all mitochondrial respiration traits were unaffected by acclimation temperature. Moreover, while ROUTINE and LEAK respirations, and associated flux control ratios, were strongly affected by assay temperature, body temperature was unrelated to all mitochondrial respiration traits but the E-R control efficiency (an index of respiratory reserve capacity; Gnaiger, 2020; García-Díaz et al., 2023).

Thirdly, Malkoc et al. argue that the stress status of individuals can affect both individual and mitochondrial metabolism, potentially changing the relationship between the two. While consideration of glucocorticoid levels may sometimes reveal biologically interesting relationships otherwise not apparent (Malkoc et al., 2021), other studies report that circulating stress hormone levels are unrelated to both organismal and blood cell respiration (Casagrande et al., 2023). Hence, while we agree that the physiological status of individuals, including affective state, may impact metabolism, such relationships are arguably of limited effect compared to the main issue of relating metabolic rate at different biological scales.

At any point in time, organismal metabolism can be considered a sum of mitochondrial respiration across the body (Salin et al., 2016), with some oxidative tissues contributing more heavily to the energy expenditure than others (Casagrande et al., 2023). However, even if mitochondrial metabolism affects organismal performance (Koch et al., 2021) and can thus be related to fitness (Hood et al., 2018), mitochondrial respiration differs between tissues (Casagrande et al., 2023; Salin et al., 2016, 2019), and subpopulations of distinct mitochondrial phenotypes may be present within single tissues (Scott et al., 2018). Moreover, mitochondrial metabolism is affected by multiple biotic and abiotic factors (Sokolova, 2018), and the conditions under which mitochondrial metabolism is measured, whether with intact or permeabilized cells, are always far from the physiological conditions experienced by cells within organisms (i.e. hormonal status, connection with the other tissues, substrate availability, supra-physiological oxygen concentrations, etc.). Organismal metabolic rate is equally labile when extrinsic and intrinsic conditions change (Versteegh et al., 2008; Norin and Malte, 2011; White et al., 2013; Welcker et al., 2015; Burton et al., 2011). Thus, the relationship between organismal metabolic rate and blood cell respiration remains a physiological parameter that must be interpreted with caution as prediction of RMR from mitochondrial respiration in specific tissues may be imprecise (Thoral et al., 2024).

To uncover the ecological and mechanistic explanations for why there is a relationship between blood cell respiration and RMR, we believe that future studies would benefit from addressing: 1) the relative aerobic level of bird blood, by estimating the proportion of ATP produced via the oxidative and glycolytic pathways compared to the situation in other tissues; and 2), as already initiated by Casagrande et al. (2023), partitioning the total contributions of mitochondrial respiration across key metabolic tissues towards RMR, when substrate availability, environmental conditions, and affective state of individuals vary; and 3) determining whether the *response* to thermal acclimation over short and long time periods is comparable between blood cells and tissues with more clearly defined roles in energy metabolism and thermogenesis.
